# GC-MS-Based Metabolomics for the Smut Fungus *Ustilago maydis*: A Comprehensive Method Optimization to Quantify Intracellular Metabolites

**DOI:** 10.3389/fmolb.2020.00211

**Published:** 2020-08-19

**Authors:** An N. T. Phan, Lars M. Blank

**Affiliations:** Institute of Applied Microbiology – iAMB, Aachen Biology and Biotechnology – ABBt, RWTH Aachen University, Aachen, Germany

**Keywords:** metabolomics, GC-MS/MS, *Ustilago maydis*, sample preparation, *Ustilaginaceae*, metabolic engineering

## Abstract

*Ustilago maydis*, a smut fungus, is an appealing model in fundamental research and an upcoming cell factory for industrial biotechnology. The genome of *U. maydis* has been sequenced and some synthesis pathways were biochemically described; however, the operation of the cellular metabolic network is not well-characterized. Thus, we conducted a comprehensive study to optimize the sample preparation procedure for metabolomics of *U. maydis* using GC-MS/MS. Due to the unique characteristics of *U. maydis* cell culture, two quenching solutions, different washing steps, eight extraction methods, and three derivatization conditions have been examined. The optimal method was then applied for stable isotope-assisted quantification of low molecular weight hydrophilic metabolites while *U. maydis* utilized different carbon sources including sucrose, glucose, and fructose. This study is the first report on a methodology for absolute quantification of intracellular metabolites in *U. maydis* central carbon metabolism such as sugars, sugar phosphates, organic acids, amino acids, and nucleotides. For biotechnological use, this method is crucial to exploit the full production potential of this fungus and can also be used to study other fungi of the family *Ustilaginaceae*.

## Introduction

*Ustilago maydis* is a maize pathogen that causes corn smut (Brefort et al., 2009), a plant disease reducing cereal production. *U. maydis* has a long history as a model organism for the study of pathogen-host interactions, fungal mating, DNA recombination, and DNA repair (Bölker, 2001; Martinez-Espinoza et al., 2002; Matei and Doehlemann, 2016). Genome sequencing (Kämper et al., 2006) revealed that approximately 10% of *U. maydis* proteins were highly conserved in humans, which had lower similarity or did not even exist in the yeast *Saccharomyces cerevisiae* (Münsterkötter and Steinberg, 2007). This finding makes *U. maydis* a unique model organism for many fundamental studies that yeast models do not offer such as endocytosis, long-distance mRNA transport, cell signaling, microtubule organization, and polarized growth (Steinberg and Perez-Martin, 2008; Etxebeste and Espeso, 2016; Haag et al., 2019).

In addition, *U. maydis*, which grows in its haploid form non-filamentous (Kubicek et al., 2011), gains attention in biotechnology due to its capability of using sustainable substrates to produce valuable chemicals of industrial interest such as mannitol, erythritol, mannosylerythritol lipids (MELs), ustilagic acid, itaconic acid, malic acid, and hydroxyparaconic acid (Geiser et al., 2014). A broad range of molecular, genetic, bioinformatic, and cell biological techniques have been developed to take full advantage of this model organism (Steinberg and Perez-Martin, 2008; Khrunyk et al., 2010; Schuster et al., 2016). “Omics” studies including genomics, transcriptomics, proteomics, and metabolomics together can elucidate cell physiology even further. The methodologies for comparative genome analysis (Schirawski et al., 2010; Laurie et al., 2012), transcriptomics (Doyle et al., 2011; Islamovic et al., 2015; Donaldson et al., 2017), and proteomics (Böhmer et al., 2007; Martínez-Salgado et al., 2013) have been established. However, a well-characterized metabolomics method is missing and, indeed, only a few biochemical synthesis pathways with a very limited number of detected metabolites were reported (Hewald et al., 2006; Teichmann et al., 2010; Winterberg et al., 2010; Jonkers et al., 2012). Hence, to study the genotype-phenotype relationship in *U. maydis*, it is crucial to develop a robust protocol for metabolomics.

There is no universal sample preparation in metabolomics that can be applied to all types of cells. The procedure may vary depending on cell properties including cell size, morphology in general, cell wall structure, but also growth conditions, growth rate, and metabolome complexity (Gulik et al., 2013), and not at least on the analytical method of choice. Moreover, *Ustilaginaceae* are well known for the ability to tolerate high substrate concentrations, grow in high cell density, and produce high titer of extracellular products (Zambanini et al., 2016; Becker et al., 2020). Therefore, it is very challenging to isolate the cells from a viscous and complex medium to obtain reliable information on the metabolome.

Here, we focused on metabolomics using gas chromatography coupled with mass spectrometry (GC-MS/MS), one of the analytical methods that covers a wide range of primary metabolites in a single run (Schauer et al., 2005; Oldiges et al., 2007; Fiehn, 2016). Rapid sampling is critical for metabolomics, as especially metabolites from central carbon metabolism have fast turnover rates (Buchholz et al., 2002). There are several methods to stop microbial cell metabolism including fast filtration, and quick quenching with organic solvents or cryoprotectants (Pinu et al., 2017). Additional washing steps using a cold biological buffer such as saline buffer or phosphate-buffered saline can be added to remove analyte-rich culture medium. Notably, glycerol, the most popular cryoprotectant, is not applicable in this case because it intrudes on the derivatization for GC-MS analysis. Next, a proper extraction procedure is necessary to release the metabolites from the cells. Extraction solvents with different polarities (i.e., water, ethanol, methanol, or solvent mixtures) are usually employed in combination with physical forces (i.e., temperature, sonication, vortex or microwave) to enhance extraction efficiency (Pinu et al., 2017). The metabolite compositions obtained after extraction have an impact on sample derivatization (Kanani et al., 2008). For the two-step method using methoximation followed by silylation, there are various conditions with different temperatures and incubation times ranging between 30–90°C and from 0.5–6 h, respectively (Moros et al., 2016). Effective sample preparation methods have been developed for many fungi such as *S. cerevisiae*, *Aspergillus* sp., *Monascus ruber*, and *Penicillium* sp. (Hajjaj et al., 1998; Jernejc, 2004; Canelas et al., 2009; de Jonge et al., 2012; Duportet et al., 2012; Kim et al., 2013; Zheng et al., 2019). However, to our knowledge, no metabolomics studies have been performed using *U. maydis*.

In this study, we aimed to optimize every step of sample preparation including quenching, washing, extraction, and derivatization for metabolomics of *U. maydis.* The method was then applied for absolute quantification of intracellular metabolites using an isotope-assisted approach, which supports deciphering the metabolic network operation in *U. maydis.* Hence, the results from the present study will contribute to the ever-increasing toolbox that makes *U. maydis* a model organism. In addition, the method presented can be used for metabolomics studies of other fungi of the family *Ustilaginaceae*.

## Materials and Methods

### Strain and Culture Conditions

*Ustilago maydis* strain MB215 (DSM17144) was used for all experiments. MTM medium was used according to Geiser et al. (2014) with 50 g L^–1^ glucose, 100 mM 2-(*N*-morpholino)ethanesulfonic acid (MES), 0.2 g L^–1^ MgSO_4_⋅7H_2_O, 10 mg L^–1^ FeSO_4_⋅7H_2_O, 0.5 g L^–1^ KH_2_PO_4_, 0.8 g L^–1^ NH_4_Cl, 1 mL L^–1^ vitamin solution and 1 mL L^–1^ trace element solution. For the experiment using different carbon sources, the same concentration (g/L) of fructose or sucrose were utilized instead of glucose. Experiments were performed in 24-deep well plates (*Enzyscreen*, System Duetz^®^) (Duetz et al., 2000) with 1.5 mL MTM per well, incubated at 30°C, relative air humidity of 80% and shaking speed of 300 rpm (*Infors HT Multitron Pro* shaker).

### Sample Collection

Main cultures were started at OD_600_ of 0.5 and OD_600_ was checked over time to determine the collection time. At OD_600_ of 20, corresponding to the mid-exponential phase of cell growth, a volume equal to 10 OD_600_ unit were collected for intracellular metabolite measurement. The same volume of samples was transferred to pre-weighed dry Eppendorf tubes to determine cell dry weight (CDW). The samples were washed twice with water and then dried in an oven at 70°C until a constant weight was achieved.

### Quenching and Washing Conditions

Nylon filters with pore sizes of 0.2 and 0.45 μm (hydrophilic, 25 mm diameter, *Millipore*) were used for fast filtration. For quick quenching, two solutions were examined including 4°C Saline Buffer 0.9% NaCl (SB) and −20°C absolute methanol (MeOH). The broth culture was cast into the precooled quenching solution at a ratio of l: 6 and mixed well. The mixture was centrifuged at 12,000 rpm for 30 s. The pellet was washed 1–3 times with 1.5 mL SB. Before determining the optimal extraction conditions, a common extraction method using chloroform/MeOH/water (2:5:2) was applied (Dempo et al., 2014; Hashim et al., 2014; An et al., 2017).

### ATP Measurement and Leakage Calculation

The level of ATP in quenching and washing solution were directly measured using the Molecular Probes^®^ ATP Determination Kit (Invitrogen, Thermo Fisher Scientific). The percentage of ATP leakage was defined by the ratio of extracellular [ATP] to total [ATP] (extracellular + intracellular). We could not determine adenylate energy charge due to the limitation of analytical methods. Instead, we combined the ATP-leakage with peak RSD-values to evaluate intracellular metabolite leakages and method reproducibility.

### Extraction Methods

Three extraction solutions were tested including ethanol (EtOH: H_2_O; 7:2), MeOH (MeOH: H_2_O; 7:2), and chloroform/MeOH/water mixture (2:5:2). In total, 900 μL of extract solution was used for each sample and ribitol was used as an internal standard with the final concentration of 20 μM. Hot extraction (95°C for 5 min and 4°C for 5 min) was applied to EtOH (HE) and MeOH (HM). Cold extraction (vortexed at 4°C for 1 h) was applied to EtOH (CE), MeOH (CM), and chloroform/MeOH/water mixture (CC). Sonication (sonicated for 30 s and set on ice for 2 min, repeat 3 times) was applied to EtOH (SE), MeOH (SM), and chloroform/MeOH/water mixture (SC). Then, all samples were centrifuged (13,000 rpm for 5 min) to collect the supernatant. When using chloroform/MeOH/water mixture to extract metabolites, 900 μL supernatant was mixed with 400 μL water and then centrifuged to separate the polar and non-polar phases. 400 μL of the polar phase were transferred into a fresh 1.5-mL tube. For other methods, 400 μL supernatant was directly transferred to a 1.5-mL microtube. Finally, the solvent was removed using a centrifugal concentrator. In our study, the matrix effects from the various extraction protocols were not examined as matrix effects are generally neglected in GC-MS and we did not encounter problems with the background signal or with peak separation.

### Metabolite Derivatization

50 μL of methoxyamine hydrochloride (MeOX) (20 mg/mL in pyridine, *Sigma Aldrich*) were added to each sample for methoximation. Samples were incubated at 37°C (for 90 min), 60°C (for 30 min), or 80°C (for 30 min). Then, silylation has been performed by adding 50 μL of *N*-methyl-*N*-(trimethylsilyl) trifluoroacetamide (MSTFA) (*Sigma Aldrich*) and incubated at 37, 60, or 80°C for 30 min. Before examining the optimal derivatization condition, all samples were derivatized at 37°C.

### GC-MS/MS Method

The analysis was performed on Trace GC Ultra coupled with TSQ 8000 (*Thermo Fisher Scientific*). Tuning and calibration were done before analysis. A 1 μL aliquot of the derivatives was injected into a VF-5 ms capillary column (30 m × 250 μm i.d., 0.25 μm film thickness) with 10 m EZ-Guard (*Agilent*). Each sample was injected twice with two different split modes including 20:1 (v/v) for low concentration metabolites and 100:1 (v/v) for high concentration metabolites. The injection temperature was 250°C and the helium carrier gas flow was 1 mL/min. The column temperature was held at 100°C for 2 min, increased by 15°C/min to 200°C (held for 4 min) and then increased by 20°C/min to 325°C (held for 5 min). The transfer line 280°C and ion source temperatures were 300°C. Ions were generated by electron ionization (EI) at 70 eV and analyzed in positive mode. Argon was utilized as a collision gas.

### Data Processing

A Selected Reaction Monitoring (SRM) transition library of metabolites was constructed using authentic standards from *Sigma-Aldrich* (Supplementary Table S1). Standard mixtures were injected periodically throughout the analysis to evaluate the stability of the analytical system as well as to support peak identification. All data were processed by using Thermo Xcalibur software version 3.1 (*Thermo Scientific*).

*Relative quantification* was used during method optimization (Supplementary Tables S2–S4). All peak areas obtained from GC-MS/MS analysis were normalized against the ribitol signal.

*Absolute quantification* was applied to measure intracellular metabolite levels of *U. maydis* while using different carbon sources (Supplementary Table S5). The extract from *U. maydis* grown in fully U-^13^C-labeled glucose (*Sigma-Aldrich*) was used as internal standard. The absolute concentrations of intracellular metabolites in the samples were calculated by using ^12^C/^13^C ratio-based calibration curves.

### Statistical Analyses

Relative standard deviations (RSD) of all detected peaks were calculated after normalization. Unit Variance (UV) scale was applied for all datasets prior to statistical analyses. Principle Component Analysis (PCA) was performed utilizing SIMCA-P^+^ version 15.0.2 (Umetrics, Umea). Hierarchical Cluster Analysis (HCA), *t*-test, one-way ANOVA test, and pathway enrichment analysis were performed using MetaboAnalyst 4.0 (Chong et al., 2018).

All detected metabolites were used for PCAs. Only metabolites that were significantly different in each condition (*p*-values < 0.05) were employed for HCAs. HCAs were calculated based on the Euclidean correlation with the Ward clustering algorithm. In *t*-test and one-way ANOVA test, *p*-value adjustments for multiple metabolites were carried out by using false discovery rate (FDR) adjustments.

## Results and Discussion

### Metabolite Leakage During Quenching and Washing

While quenching is crucial to stop cell metabolism quicker than the turnover of metabolites, washing is utilized to remove extracellular compounds from the culture medium. These steps have to compromise to keep the metabolites lost at a minimum. First, we tested fast filtration using filters with pore sizes of 0.2 and 0.45 μm. Because of sample viscosity, the cells were not able to pass the membranes completely, despite vacuum was applied. This problem is most likely due to the excessive abundance of extracellular lipids, like MELs, which has been reported to cause troubles in the centrifugation efficiency of the liquid medium (Becker et al., 2020).

Quick quenching methods were subsequently examined using SB and MeOH as quenching solutions. Interestingly, *U. maydis* cells tended to accumulate when interacting with MeOH (Supplementary Figure S1). Cell accumulation interfered with further washing steps and led to the detection of mainly a saturated glucose peak as well as other extracellular product peaks. Since polysaccharides are essential components of the fungal cell wall and *U. maydis* secretes β-D-glucan (Fonseca-García et al., 2011), the observed phenomenon might be a result of polysaccharide precipitation in organic solvents. Thus, SB with better performance and less leakage was chosen as not only quenching but also washing solution in further experiments (Figure 2A).

We evaluated each optimization step based on two main criteria. First, the reproducibility was assessed based on RSD values. A common standard for a good method is an RSD value of detected peak of lower than 30%. Among metabolites in central metabolic pathways, it is always challenging to detect phosphorylated compounds. Therefore, the second priority is for the method that is able to measure this group of metabolites. As predicted, the use of SB, a biological buffer, did not lead to severe metabolite leakage. The leak percentage increased when more washing steps with SB were added but none of them exceeded 3% (Figure 2B). The least robust condition was washing only one time with SB (Figures 2C,D). Approximately one-third of detected peaks had RSD values higher than 30% and the levels of many phosphorylated metabolites were significantly lower. The lower sensitivity might occur due to the remaining extracellular compounds, which affected the derivatization efficiency. Washing two times with SB appealed to be the best condition because of the high reproducibility of overall metabolite measurements and the determination of phosphorylated compounds.

### Extraction Methods for Low Molecular Weight Hydrophilic Metabolites in Central Metabolic Pathways

**FIGURE 1:**
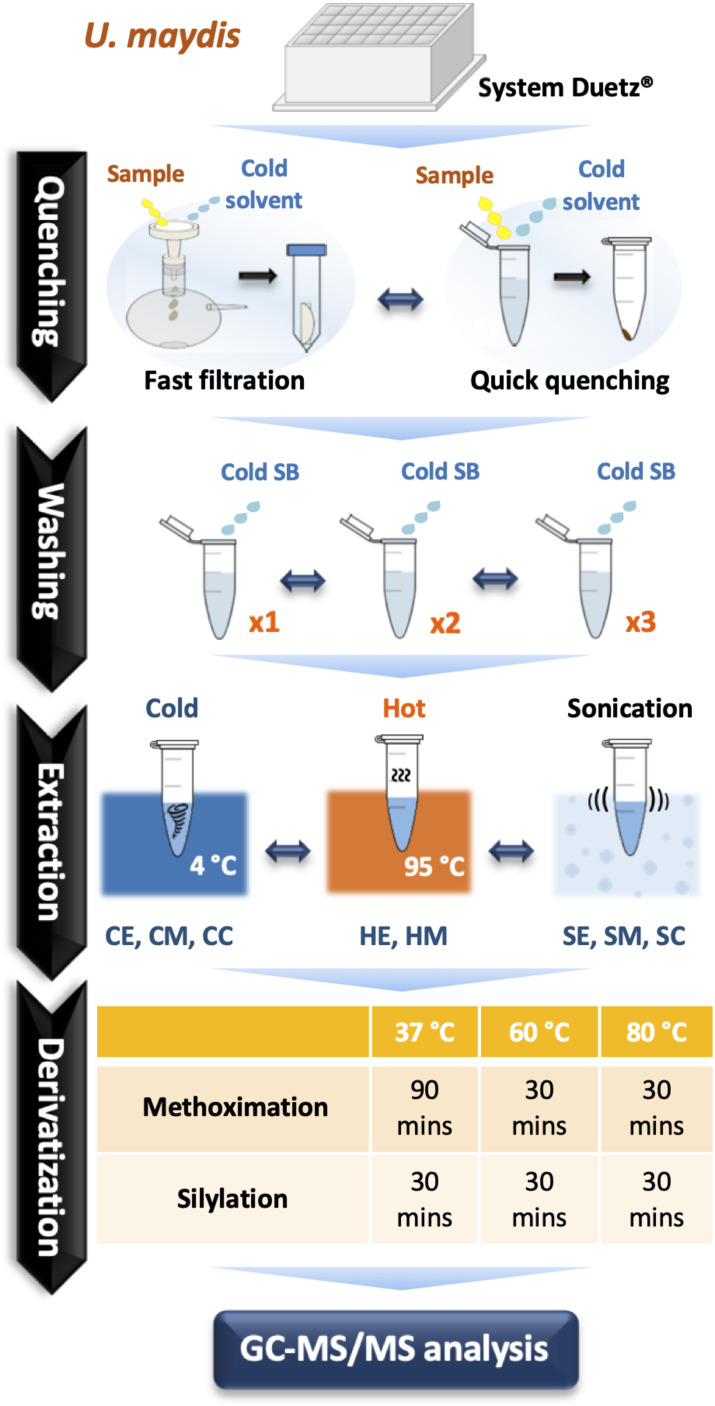
A comprehensive optimization of sample preparation for GC-MS/MS-based metabolomics in *U. maydis*. Cell cultivations were performed in System Duetz^®^ 24-deep well plates at 30°C in minimal media. To instantly stop cellular metabolism, both fast filtration and quick quenching methods were tested. Then, cells were washed one (x1), two (x2), and three (x3) times with cold saline buffer to remove the extracellular components from culture medium. Eight different strategies combining chemical and mechanical disruption methods were tested for extraction efficiency. Finally, metabolites were derivatized at 37°C, 60°C, or 80°C. All experiments were performed with three biological replicates. Abbreviations: SB, Saline Buffer; CE, cold EtOH; CM, cold MeOH; CC, cold chloroform/MeOH/water mixture; HE, hot EtOH; HM, hot MeOH; SE, EtOH with sonication; SM, MeOH with sonication; SC, chloroform/MeOH/water mixture with sonication.

The choice of extraction method will define the number, type, and abundance of detected metabolites from *U. maydis* cells. A proper extraction method should be able to make the cell envelope permeable to release metabolites completely and denature cellular enzymes to prevent metabolite degradation during sample preparation. Yet, the extraction condition itself should not cause metabolite degradation or transformation. In order to figure out a suitable extraction method, we combined both chemical and mechanical disruption methods into eight different strategies (Figure 1). Together with the RSD values and phosphorylated compound levels as mentioned above, PCA was applied to have an overview of the whole dataset among different extraction conditions (Figure 4, upper panels). One-way ANOVA test or *t*-test was performed to figure out the metabolites which had significantly different levels in each condition. The metabolites with adjusted *p*-values lower than 0.05 were submitted to HCA, a method to cluster the metabolites according to their abundance (Figure 4, lower panels).

**FIGURE 2:**
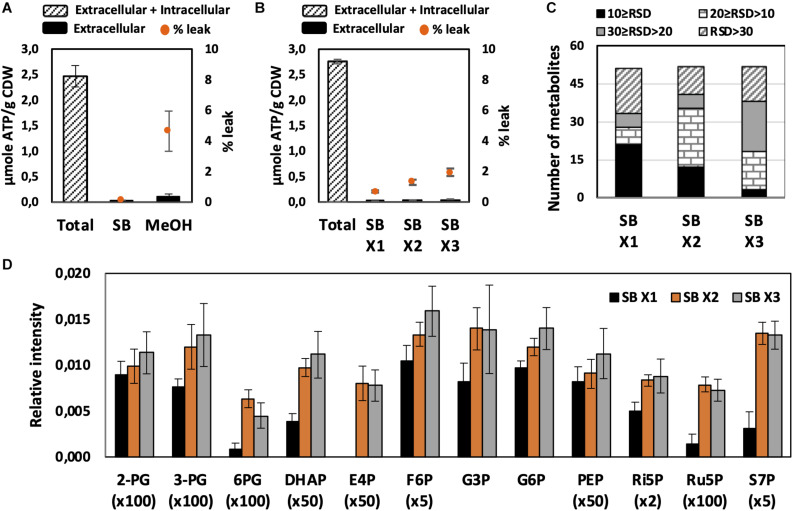
Effect of quenching and washing on *U. maydis* intracellular metabolites. **(A)** Metabolite leakage while quenching with SB and MeOH. **(B)** Metabolite leakage, **(C)** Relative standard deviations (RSD) of all detected peaks, and **(D)** phosphorylated metabolite levels when *U. maydis* cells were washed one (x1), two (x2), and three (x3) times with SB. Each data point represents the average of three biological replication with the error bar indicating the standard deviation. Abbreviations: 2-PG, 2-phosphoglycerate, 3-PG: 3-phosphoglycerate; 6PG, 6-phosphogluconate; DHAP, dihydroxyacetone phosphate; E4P, erythrose-4-phosphate; F6P, fructose-6-phosphate; G3P, glycerol-3-phosphate; G6P, glucose-6-phosphate; PEP, phosphoenolpyruvate; Ri5P, ribose-5-phosphate; Ru5P, ribulose-5-phosphate; and S7P, sedoheptulose-7-phosphate. Normalized peak intensities of some metabolites were multiplied for data visualization.

One critical point during sample preparation was that *U. maydis* cells should not interact directly to organic solvent after centrifugation. The cell pellets had to be mixed with the water portion of extraction solvents first. After the cells were fully resuspended, the organic solvents were added, and extraction started. When the pre-mixed extract solvent was used, *U. maydis* cells accumulated (as in Supplementary Figure S1) and were not able to be fully resuspended; thus, the results were not reproducible. This was also the reason why neat organic solvents were not suitable to extract intracellular metabolites from *U. maydis*.

**FIGURE 3:**
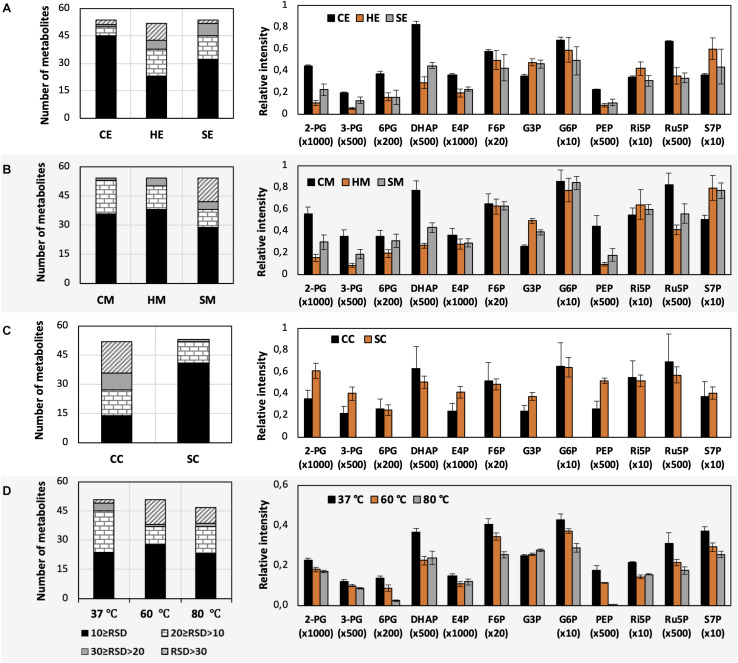
Impacts of different extraction and derivatization conditions on *U. maydis* intracellular metabolites. RSD values and the levels of phosphorylated metabolites extracted with **(A)** EtOH, **(B)** MeOH, and **(C)** chloroform/MeOH/water; as well as **(D)** derivatized under different temperatures. Each data point represents the average of three biological replication with the error bar indicating the standard deviation. Normalized peak intensities of some metabolites were multiplied for data visualization.

**FIGURE 4:**
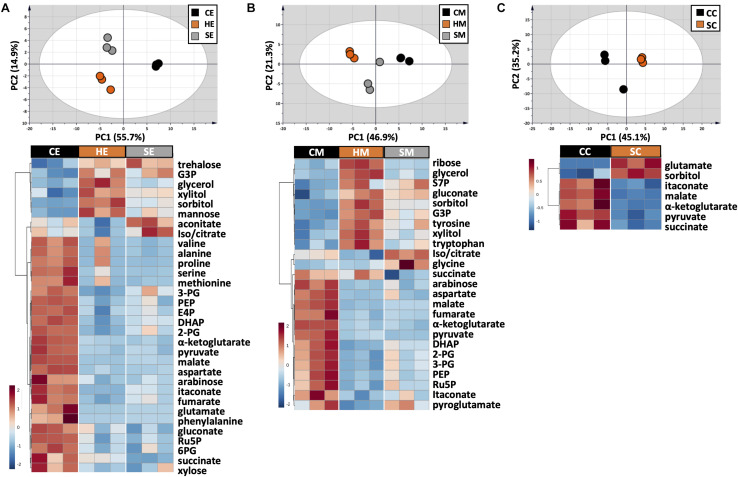
Metabolic profile comparison during extraction optimization. Upper panels are PCA score plots and lower panels are the HCA heatmaps of metabolites extracted with **(A)** EtOH, **(B)** MeOH, and **(C)** a chloroform/MeOH/water mixture. While the whole dataset was used for PCA, only metabolites with adjusted *p*-value < 0.05 were used for HCA. Each experiment consisted of three biological replications and UV scale was applied prior to statistical analyses. The color scales in HCA indicate relative intensity after normalization and scaling.

The use of MeOH and EtOH resulted in very comparable results under all tested conditions, which was reasonable as these organic solvents have very close polarity indices of 5.1 and 5.2, respectively (Ramluckan et al., 2014). Cold extraction showed the highest reproducibility. Almost every metabolite had RSD values lower than 30% (Figures 3A,B). Changing in extraction conditions from hot to sonicate, and to cold methods showed increased levels of phosphorylated metabolites, especially for low abundant compounds such as 2-PG, 3-PG, 6PG, DHAP, E4P, PEP, and Ru5P (Figures 3A,B). Moreover, the organic acids including pyruvate, malate, succinate, α-ketoglutarate, and itaconate could be extracted better with CE and CM (Figures 4A,B). Low metabolite levels in hot extraction were most likely due to their thermolability.

The method using chloroform/MeOH/water is very popular for the extraction of both polar and non-polar metabolites. Here, we found that this solvent mixture could extract *U. maydis* metabolites better with sonication (SC) than vortex at low temperature (CC). The overall reproducibility was drastically improved in SC, even though the intensities for most of the metabolites were not significantly different between CC and SC (Figures 3C, 4C). Vice versa, the use of sonication for EtOH and MeOH was not as good as cold extraction. While sonication is more aggressive to cell membranes comparing to vortexing, alcohols cannot denature protein as good as chloroform (Asakura et al., 1978). Some enzymes might still be partly active during SE and SM; thus, affected metabolite stability.

Generally, the three extraction conditions CE, CM, and SC were all suitable for GC-MS/MS-based metabolomics of *U. maydis*, with an overall good reproducibility (Supplementary Figure S2). In addition, the sensitivity was reasonable as all metabolites were in the quantification range of GC-MS/MS, especially for phosphorylated metabolites. For the next steps, CE was chosen because EtOH is environmentally friendly, not carcinogenic, and sample preparation for CE was simple.

### Effects of Derivation Conditions on GC-MS/MS Analysis

Almost every metabolite investigated requires derivatization to increase volatility for GC-MS analysis. The two-step derivatization method using methoximation and silylation is well established in the field of metabolomics (Fiehn, 2016). First, MeOX reacts to metabolites with ketone groups. This step is important to open sugar rings, which results in fewer peaks per sugar in GC-MS analysis (Yi et al., 2014). MSTFA is used to derivatize chemical components with an active H, such as –OH, –COOH, –NH_2_, and –SH. Previous studies have already shown that the formation and stability of derivatives among metabolites differ greatly.

**FIGURE 5:**
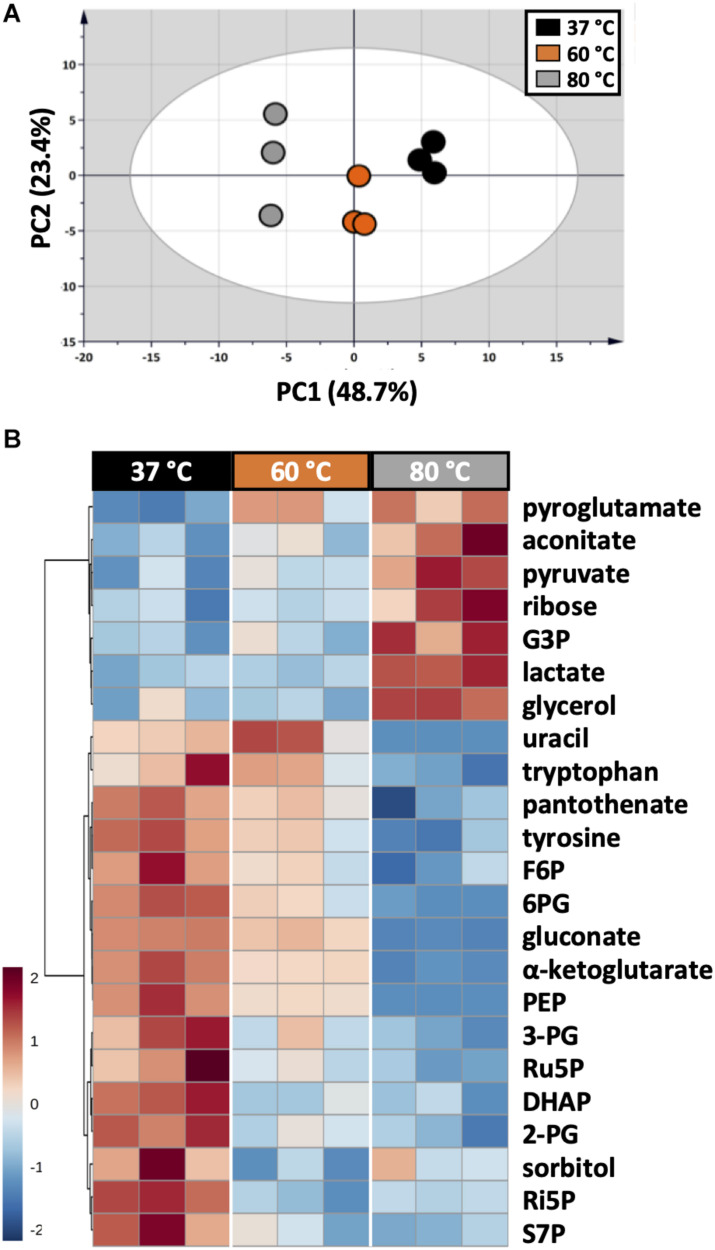
Impacts of different derivatization conditions on *U. maydis* intracellular metabolites. **(A)** PCA score plot acquired from the complete dataset. **(B)** HCA heatmap of metabolites with adjusted *p*-value < 0.05 from one-way ANOVA test. Each experiment had three biological replicates and UV scale was applied before statistical analyses. The color scale in HCA indicates relative intensity after normalization and scaling.

As each organism has unique metabolic profiles, derivatization conditions should be studied prior to applications. Here, three conditions commonly used to derivatize metabolites from central carbon metabolism were examined (Figure 1). Throughout derivatization, metabolites were exposed to high temperature for at least 1 h. The observed RSD values indicated that the higher the temperature, the more unstable many metabolites were (Figure 3D). The signals of almost all metabolites were highest when derivatized at 37°C (Figure 5). Moreover, the peaks of proline and phenylalanine could not be detected after derivatization at 80°C. This result was consistent with previous results from the extraction step, showing that primary metabolites were sensitive to high temperatures. Hence, we recommend performing both methoximation and silylation at 37°C.

### Absolute Quantification of Intracellular Metabolites When *U. maydis* Is Grown on Different Carbon Sources

The fungal family *Ustilaginaceae* is well-known for the capability of using a board range of carbon sources to produce molecules of industrial interest such as organic acids, glycolipids, and sugar alcohols (Geiser et al., 2014). The optimized sample preparation method was employed to measure intracellular metabolite levels while *U. maydis* utilizing sucrose, glucose, and fructose as sole carbon source. In order to apply the isotope-assisted approach for absolute quantification, the extract from *U. maydis* grown in fully U-^13^C-labeled glucose was used as an internal standard.

**FIGURE 6:**
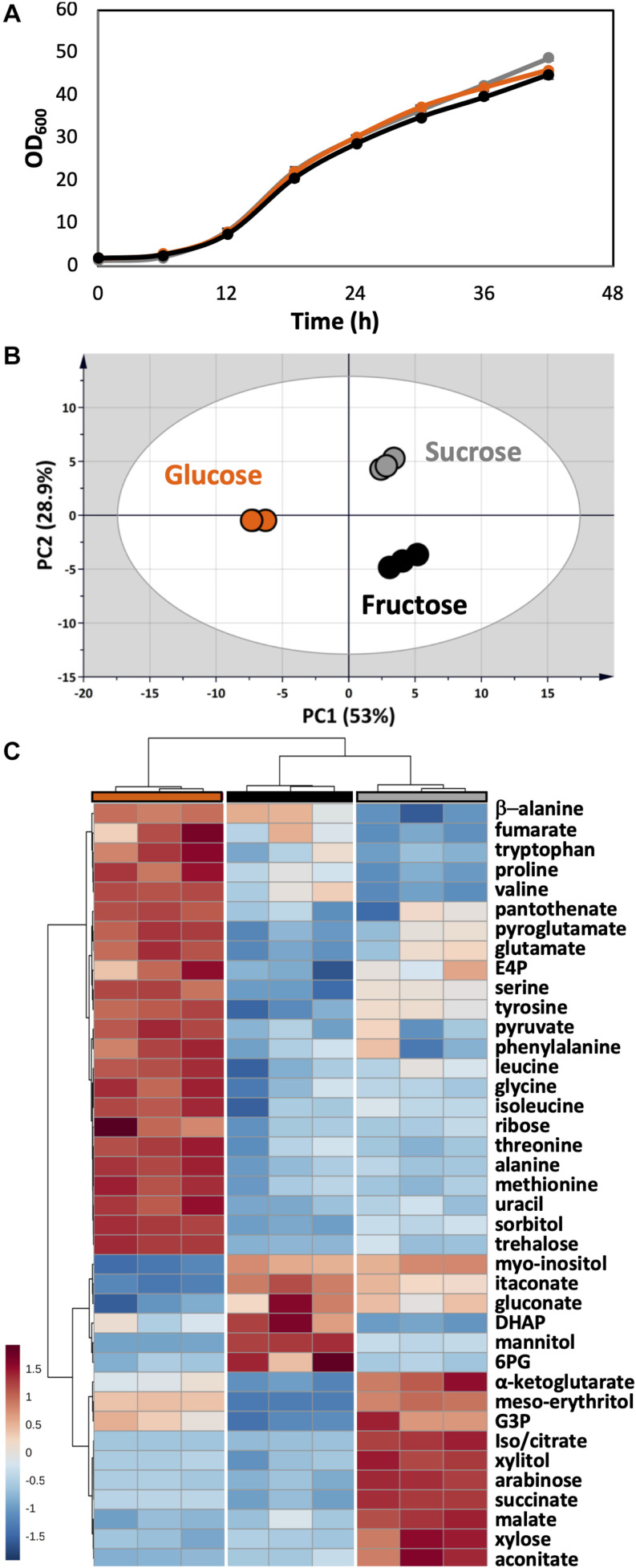
Metabolome analysis of *U. maydis* during growth with glucose, fructose, and sucrose as sole carbon sources. **(A)** Growth curves were determined by measuring OD_600_. **(B)** PCA was conducted with all detected metabolites. **(C)** The HCA heatmap indicates significant different metabolites among all conditions. The colors were consistent among each group of samples: glucose (orange), fructose (black), and sucrose (gray). All experiments were performed with three biological replicates.

As a plant pathogen, *U. maydis* had no problem in using fructose and sucrose. The growth of *U. maydis* on these carbon sources was very similar when compared with growth on glucose (Figure 6A). Though, cells grown on different carbon sources had distinct metabolic profiles, indicated by three clearly separated groups in the PCA score plot (Figure 6B). Samples in the “glucose” group were most distinct from the other samples, shown in the high separation on PC1 (46.6%) in the PCA and the distance in HCA clusters (Figures 6B,C). The one-way ANOVA test scored 39 out of 51 metabolite concentrations as significantly different with an adjusted *p*-value < 0.05.

The concentrations of free amino acids were high when *U. maydis* used glucose. Especially, the high abundances of metabolites related to aromatic amino acid metabolism including erythritol, E4P, phenylalanine, tyrosine, and tryptophan was observed. On the other hand, a high level of mannitol was found when *U. maydis* utilized fructose, most likely because fructose can be converted directly to mannitol by the mannitol dehydrogenase. The concentrations of many organic acids of the TCA cycle were divided into two sets while comparing “glucose” and “fructose” groups. Acotinate and itaconate levels were higher in the “fructose” group; however, pyruvate, malate, fumarate, succinate, and α-ketoglutarate concentrations were higher in the “glucose” group.

From the annotated genome, *U. maydis* has a putative invertase (UMAG_01945), which is potentially capable of hydrolyzing sucrose into glucose and fructose molecules (Kämper et al., 2006). Thus, the metabolite compositions of the “sucrose” group shared the features of both “glucose” and “fructose” groups. While the concentration of intracellular mannitol was higher than cells grown on glucose, the level of erythritol was the highest among all conditions.

Together, the choice of carbon source had substantial impact on intracellular metabolite concentrations. Depending on the target compound, metabolic engineering can be employed to alter the metabolic flux distribution to maximize product synthesis. Erythritol and mannitol are well-known as sweeteners (Grembecka, 2015). The carboxylic acids in the TCA cycle are listed among the value-added chemicals from biomass (Werpy and Petersen, 2004). Amino acids are important dietary supplements of mainly animals, the aromatic amino acids can be used as precursors for industrial and pharmaceutical compounds (i.e., aspartame, L-DOPA, melanin, or phenol) (Parthasarathy et al., 2018; Wynands et al., 2018).

For the first time, the absolute quantification of intracellular metabolites was conducted in *U. maydis*. The present study provides a well-established sample preparation method for GC-MS/MS-based metabolomics, which still can be further adjusted according to research objectives or to a new analytical device (i.e., nuclear magnetic resonance or other MS-based equipment). This method will further support the development of *U. maydis* from a basic science model organism, to a next-generation model organism also fulfilling the requirements for modern biotechnology.

## Data Availability Statement

All datasets presented in this study are included in the article/Supplementary Material.

## Author Contributions

AP performed all the experiments. LB supervised the study. Both authors analyzed the data. Both authors have approved the final version of the manuscript.

## Conflict of Interest

The authors declare that the research was conducted in the absence of any commercial or financial relationships that could be construed as a potential conflict of interest.

## Supplementary Material

FIGURE S1 *Ustilago maydis* cells in quenching solution.

FIGURE S2 Comparison between CE, CM, and SC extraction method.

TABLE S1 Selected reaction monitoring (SRM) transitions for GC-MS/MS analysis.

TABLE S2 Metabolite levels while applying different washing steps.

TABLE S3 Metabolite levels and results of statistical analyses while applying different washing steps.

TABLE S4 Metabolite levels and results of statistical analyses in various derivatization conditions.

TABLE S5 Metabolite concentrations and results of statistical analyses during growth of *U. maydis* on glucose, fructose, or sucrose.
