# Supplementary material for: GC-MS-Based Metabolomics for the Smut Fungus Ustilago maydis: A Comprehensive Method Optimization to Quantify Intracellular Metabolites
Source: Front Mol Biosci. 2020 Aug 19;7:211. doi: 10.3389/fmolb.2020.00211 (PMC7468419; doi:10.3389/fmolb.2020.00211)
Supplement: FIGURE S1 — Ustilago maydis cells in quenching solution. [file Image_1.pdf]

GC-MS-based metabolomics for the smut fungus *Ustilago maydis:* a comprehensive method optimization to quantify intracellular metabolites

An N.T. Phan [1, *], Lars M. Blank [1, *]

[1] Institute of Applied Microbiology - iAMB, Aachen Biology and Biotechnology – ABBt, RWTH Aachen University, Worringerweg 1, 52074 Aachen, Germany.

Table of content

Figure S1. *U. maydis* cells in quenching solution.

Figure S2. Comparison between CE, CM and SC extraction method.

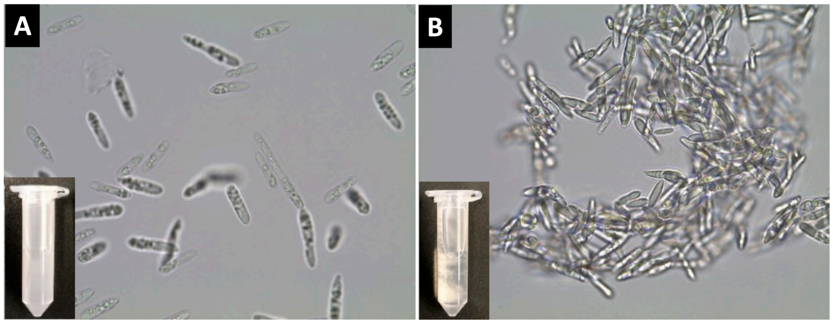

Figure S1. *U. maydis* cells in quenching solution. (A) Saline Buffer, (B) MeOH. The cells accumulated in MeOH but not in SB. The pictures were taken under a microscope with the magnification of 1000 times.

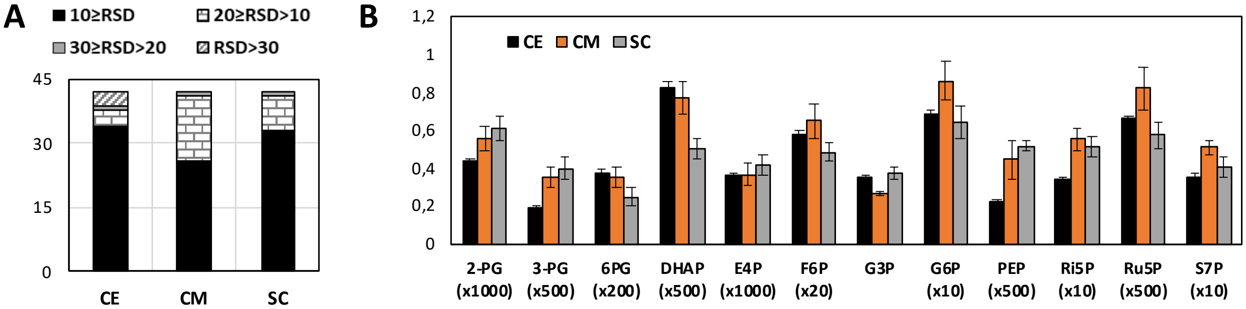

Figure S2. Comparison between CE, CM and SC extraction method. (A) RSD values and (B) relative abundance of phosphorylated metabolites. Each data point represents the average of three biological replication with the error bar indicating the standard deviation. Normalized peak intensities of some metabolites were multiplied for data visualization
